# Identification of S100A9 as Biomarker of Responsiveness to the Methotrexate/Etanercept Combination in Rheumatoid Arthritis Using a Proteomic Approach

**DOI:** 10.1371/journal.pone.0115800

**Published:** 2014-12-29

**Authors:** Antoine Obry, Thierry Lequerré, Julie Hardouin, Olivier Boyer, Patrice Fardellone, Peggy Philippe, Xavier Le Loët, Pascal Cosette, Olivier Vittecoq

**Affiliations:** 1 INSERM, U905, Pathophysiology and Biotherapy of Inflammatory and Autoimmune Diseases, F-76000 Rouen, France; 2 CNRS, UMR 6270, Polymers, Biopolymers and Surfaces, F-76821 Mont Saint Aignan, France; 3 PISSARO Proteomics Facility, F-76821 Mont Saint Aignan, France; 4 Normandy University, Institute of Research and Innovation in Biomedecine, F-76821 Mont Saint Aignan, France; 5 Department of Rheumatology, Rouen University Hospital, F-76000 Rouen, France; 6 INSERM, Centre d'investigation clinique 1404, F-76000 Rouen, France; 7 Department of Immunology, Rouen University Hospital, F-76000 Rouen, France; 8 Department of Rheumatology, Amiens University Hospital, F-80000 Amiens Cedex 1, France; 9 of Rheumatology, University Hospital of Lille, F-59037 Lille Cedex, France; Hormel Institute, University of Minnesota, United States of America

## Abstract

**Objectives:**

One way to optimize the drug prescription in rheumatoid arthritis (RA) is to identify predictive biomarkers of drug responsiveness. Here, we investigated the potential "theranostic" value of proteins of the S100 family by monitoring levels of both S100A8 and S100A9 in blood samples from RA patients.

**Design:**

For proteomic analysis, peripheral blood mononuclear cells (PBMC) and serum samples were collected in patients prior to initiation of the methotrexate/etanercept (MTX/ETA) combination. Firstly, relative mass spectrometry (MS) quantification focusing on S100A8 and S100A9 proteins was carried out from PBMCs samples to identify potential biomarkers. The same approach was also performed from serum samples from responder (R) and non responder (NR) patients. Finally, to confirm these results, an absolute quantification of S100A8, S100A9 proteins and calprotectin (heterodimer of S100A8/S100A9) was carried out on the serum samples using ELISA.

**Results:**

MS analyses revealed that both S100A8 and S100A9 proteins were significantly accumulated in PBMC from responders. In contrast to PBMC, only the S100A9 protein was significantly overexpressed in the serum of R patients. Absolute quantification by ELISA confirmed this result and pointed out a similar expression level of S100A8 protein and calprotectin in sera from both R and NR groups. Thus, the S100A9 protein revealed to be predictive of MTX/ETA responsiveness, contrarily to parameters of inflammation and auto-antibodies which did not allow significant discrimination.

**Conclusion:**

This is the first report of an overexpression of S100A9 protein in both PBMCs and serum of patients with subsequent response to the MTX/ETA combination. This protein thus represents an interesting biomarker candidate of therapeutic response in RA.

## Introduction

Rheumatoid arthritis (RA) is a chronic, autoimmune disease that results in progressive structural damage and disability. The pro-inflammatory cytokine TNF-α is a key mediator in the RA pathogenesis [1], and is as such, considered as a major therapeutic target. Indeed, five TNF blocking agents (TBAs) are now available and used as a second line of therapy after inadequate response to methotrexate. However, even if these biologic agents have improved RA patient care [2], [3], [4], 30% of them do not respond to these innovative biotherapies [5]. Noteworthy, this lack of efficacy is associated to side-effects and overcosts.

All biological agents have similar clinical and structural efficacy as well as a comparable safety profile when administered in combination with MTX. Thus, the choice of the first biological agent is difficult in practice, and particularly since almost all of them can be used as first line biotherapy after failure of at least one non-biological disease modifying anti-rheumatic drugs (DMARDs) including MTX. Considering all these issues, predicting the patient's response to a given treatment has become a very important challenge. Until now, several diagnostic and prognostic markers have been evaluated as well as a panel of soluble biomarkers derived from RA pathophysiology [6] and of candidate gene polymorphisms. However, it remains yet difficult to get valuable markers that would predict the drug responsiveness ("theranostic" biomarkers).

Even though data from studies having investigated combination of candidate proteins derived from RA pathophysiology were more encouraging, they were not validated in independent cohorts of RA patients. Thus, large scale genomic analyses seem more promising. To our knowledge, no study has investigated the identification of serum protein biomarkers for prediction of response to etanercept using an innovative proteomic approach without a priori.

In this context of identification of biomarker candidates, our attention was focused on the abundance of proteins of the S100 family, namely S100A9 (Calgranulin B or MRP14) and S100A8 (Calgranulin A or MRP8) in peripheral blood mononuclear cells (PBMC) from RA patients treated with methotrexate/etanercept (MTX/ETA) combination. These S100 proteins are secreted locally by activated neutrophils, and have been extensively studied in the context of RA. In 2002, Using a 2-DE approach, Sinz et al. compared the proteome of synovial fluid from various rheumatic diseases and identified the S100A9 protein only in RA patients [7]. Additionally, using a SELDI approach, other groups found the S100A8 and S100A12 (Calgranulin C, MRP6) proteins as markers able to differentiate RA from osteoarthritis [8], [9], [10]. Interestingly, S100A8 and S100A9 proteins can assemble into an heterodimer referred to as calprotectin. This heterodimer was identified as a marker of RA in the synovial fluid and in the plasma, with plasma concentrations differentiating RA from other rheumatic disease [11]. The prognostic value of these proteins has been suggested several times, both in plasma [12] and synovial fluid [13]. More recently, calprotectin was highlighted as a predictor of disease improvement, suggested by the decrease of the swollen joint number parallel to that of calprotectin level during the first weeks of treatment [14]. However, even if abundances of S100 proteins appear to be influenced by biologic agents, their theranostic value has never been demonstrated so far.

## Material and Method

### Study design

**Figure 1:**
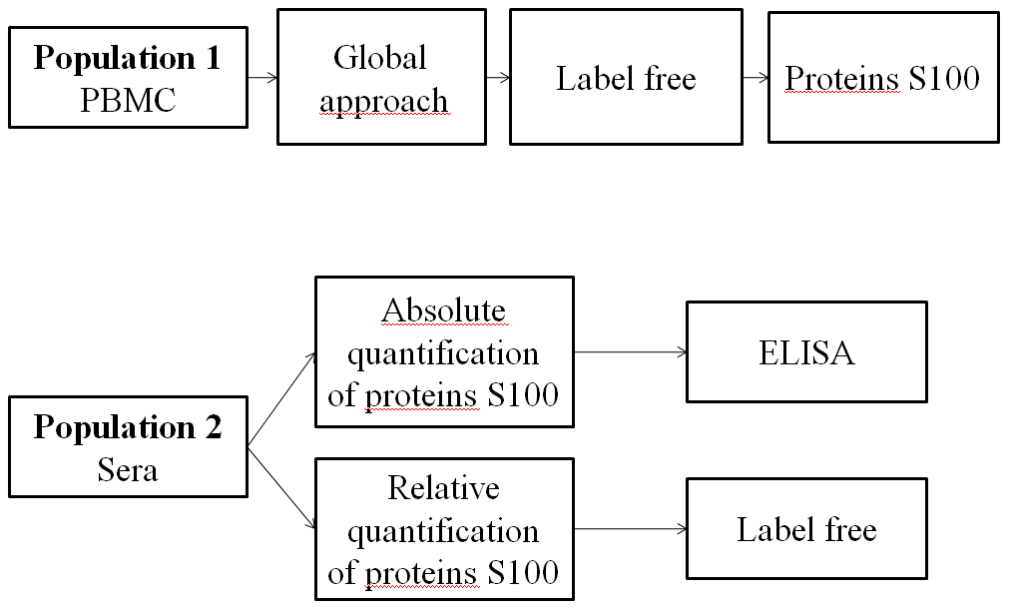
Experimental workflow. In the first stage, a label free approach was used to determine candidate proteins that are differentially expressed in PBMCs between responder (R) and non-responder (NR) RA patients before initiation of etanercept in the population 1. This approach was used to quantify the relative abundance of S100 proteins. To independently confirm these results, label free approach by mass spectrometry and absolute quantification by ELISA were used to quantify S100 proteins in sera samples from R and NR RA patient before etanercept initiation in the population 2.

In the line of previous investigations performed with transcriptomic approaches [15], we first decided to make proteomic investigations from PBMC to identify theranostic biomarkers to the MTX/ETA combination in a first RA population. This was performed by designing a relative mass spectrometry “label free” quantification [16]. We focused on the expression of S100A9 and S100A8 proteins in RA patients with active disease treated by MTX/ETA combination to determine the global expression level of those proteins. Then, we measured the abundances of S100A8 and S100A9 proteins in sera collected before treatment in a second independent cohort of RA patients. To achieve this goal, independent quantitative proteomic approaches were performed, i.e., a label free quantification and an absolute quantification by conventional ELISA. The workflow of the study is outlined in Fig. 1.

### Patients

Two cohorts of RA patients with active disease (defined by a disease activity score DAS28 >3.2), who required a biologic agent after failure of at least one non-biologic DMARD have been studied. In both cohorts, each patient has received the MTX/ETA combination at stable doses. All patients fulfilled the ACR criteria [17], were biologic DMARD naïve and received MTX at stable dose since 1 month and for some of them glucocorticoids at a dose ≤10 mg per day. The first cohort (Population 1) that included 6 RA patients was devoted to study the proteome of PBMCs while the second one (Population 2) that comprised 22 RA patients was used for quantification of S100 proteins in sera samples.

During the 12 months follow-up period, several clinical and biological parameters were collected at different time-points (i.e, 6, 12, 24 and 52 weeks) as follows: patient's assessment of pain and disease activity (using 0 to 100 mm visual analog scale), duration of morning stiffness, number of tender joints, number of swollen joints, erythrocyte sedimentation rate (ESR), C-reactive protein (CRP) level, disease activity score 28 (DAS28) and the physical function scored with the french version of the Health Assessment Questionnaire (HAQ) for RA. Levels of rheumatoid factors (RF; Rapitest^®^RF, Behring Diagnostics, Westwood, MA) and anti-cyclic citrullinated peptide antibodies (anti-CCP2; Euroimmun, GMBH, GroBGrönau, Germany) were also measured at baseline. Patients were considered RF positive if result of the Latex Fixation Test was ≥20 IU/ml and anti-CCP2 positive if titer was ≥10 AU/ml. The clinical efficacy of the MTX/ETA association was evaluated at 6 months according to the European league against rheumatism (EULAR) response criteria [18]. Accordingly, patients were categorized into good, moderate or non-responders based on the degree of change in the DAS28 and the level of DAS28 reached at 6 months. Good responders (R) were defined as patients who had a decrease in DAS28 from baseline ΔDAS28>1.2 and a DAS28 at sixth month <3.2. Moderate responders (MR) had either ΔDAS28>1.2 and a DAS28 at sixth month >3.2 or ΔDAS28 from 0.6 to 1.2 and a DAS28 at sixth month <5.1. Non-responders (NR) were those who had either ΔDAS28 <0.6 or a DAS28 at the sixth month >5.1. Only R and NR patients were included herein. During the first 6 months of follow-up, there was no change in the dose of MTX and glucocorticoids.

Sera were collected from patients included in the SATRAPE study (no. 2005/06; ClinicalTrials.gov identifier: NCT 00234234) and another study (no. 2004/120). Both studies were approved by the regional ethics committee (CPP Nord Ouest 1, France) and all participants gave written informed consent at the time of enrollment.

### Serum and PBMC samples

Blood samples were taken in the morning between 8 am and 9 am. For the first set, each sample was immediately transported at 4°C in the laboratory to remove PBMCs that were isolated from 50 ml blood samples by density gradient centrifugation over *Ficoll Unisep*. Proteins were extracted from PBMCs by sonication on ice in 1 ml of lysis buffer (7 M urea, 2 M Thiourea, 4% CHAPS, 65 mM DTT, 25 mM Tris/Cl and 100 µL of protease inhibitors), followed by centrifugation at 14,000 rpm for 10 min at 4°C. The PBMC samples were stored at −80°C during 3 years until analysis. For the second set, blood samples were allowed to coagulate and then centrifuged at 2,800 rpm/min for 10 min. The serum samples were stored at −80°C during 5 years until analysis. The protein concentrations were determined using the standard Bradford method.

### Enzymatic digestion of whole protein extracts

Twenty-five µg of proteins from sera samples were loaded on polyacrylamide gels and allowed for a short period a migration (1.5 h) in a SDS-PAGE stacking gel (7%). After staining, the revealed protein bands were excised immersed in reductive medium (5 mM dithithreitol) and cysteins were irreversibly alkylated in 25 mM iodoacetamide. After washing steps with water, gel bands were submitted to protein digestion (trypsin from Promega, 0.5 µg per band). Several steps of peptide extraction were then performed in H_2_O/CH_3_CN/TFA mixtures (50/50/1) and the peptide fractions were combined and evaporated.

### Liquid nanochromatography and mass spectrometry

For mass spectrometry analysis, peptides were dissolved in 0.1% formic acid in water. All experiments were carried out with a linear ion trap-Orbitrap mass spectrometer (LTQ Orbitrap Elite, Thermo Scientific) equipped with a nano-ESI source and coupled to a nanoliquid chromatography (Easy-nLC II, Thermo Scientific). After sample loading onto an enrichment column, the separation was achieved on a reversed phase C_18_ column (NikkyoTechnos, Japan) using a linear gradient of 15% to 55% B over 120 min (mobile phase A: H_2_O/0.1% FA; mobile phase B: ACN/0.1% FA). The elution was then directed toward the mass spectrometer with capillary voltage set at 1.5 kV. The mass spectrometer was operated in the data-dependent mode to automatically switch between Orbitrap-MS (from *m/z* 300 to 2000 with a resolution of 30,000) and LTQ-MS/MS acquisition. The mass spectrometer selected the 20 most intense ions for fragmentation.

Raw data were then imported in Progenesis LC-MS software (Nonlinear Dynamics). For comparison, one sample was set as a reference and the retention times of all other samples within the experiment were aligned. After alignment and normalization, statistical analysis was performed for one-way analysis of variance (ANOVA) calculations. For quantitation, peptide features presenting p-value <0.05 and q-value <0.05 were retained, and MS/MS spectra from selected peptides were exported for peptide identification with Mascot (Matrix Science) used against the SwissProt database restricted to Human. Peptides corresponding to S100A8 and S100A9 proteins with scores above identity threshold were considered and re-imported into Progenesis. For these proteins, the total cumulative abundance of the protein was calculated by summing the abundances of peptides. “Label free” experiments were performed in duplicate.

### ELISA

Serum levels of S100A9, S100A8 and calprotectin were determined at baseline in sera from responder (R) and non-responder (NR) patients included in the second cohort, using enzyme-linked immune-sorbent assay (ELISA) according to the manufacturer's instructions (USCNK, USA and Bluegene, China, CUSABIO, China). ELISA experiments were performed in duplicate.

### Statistical analyses

The Kolmogorov-Smirnov test was used to evaluate the data distributions. Accordingly, Mann Whitney non-parametric tests were used to compare median levels of proteins from label free experiments and ELISA. Mann Whitney non-parametric tests were also used to compare at baseline the differences of clinical and demographic data between all responders versus non-responders. To establish a relationship between dependent variables (ESR, CRP, DAS28, S100A9…) measured prior to treatment initiation and the degree of response at 6 months, univariate analyses were performed using the Spearman's rank correlation. A p-value <0.05 was considered statistically significant. These statistical analyses were carried out using GraphPad Prism 5 (GraphPad Software).

## Results

### Increased levels of S100A8 and S100A9 proteins in PBMC from responders

In the context of a global proteomic approach in PBMCs from 6 RA patients treated by the MTX/ETA combination (Population 1), we focused our attention on the abundance levels of S100A9 and S100A8 proteins that were identified as potential theranostic biomarkers among a panel of candidate proteins. Demographic, clinical and biological data of this small population have been collected and compared. According to the EULAR criteria, half of them were classified as R while the 3 other patients were NR after six months of ETA/MTX treatment. Both subsets comprised 2 women and one man. Due to the small number of patients included, no statistical analysis was carried out. Prior to treatment initiation, ESR was slightly higher in R (28.3±6.0 versus 21.3±9.6 mm/1^st^hour), and R were older (69.0±7.1 versus 53.7±8.8 years). All other parameters were fairly similar between both subgroups.

**Figure 2:**
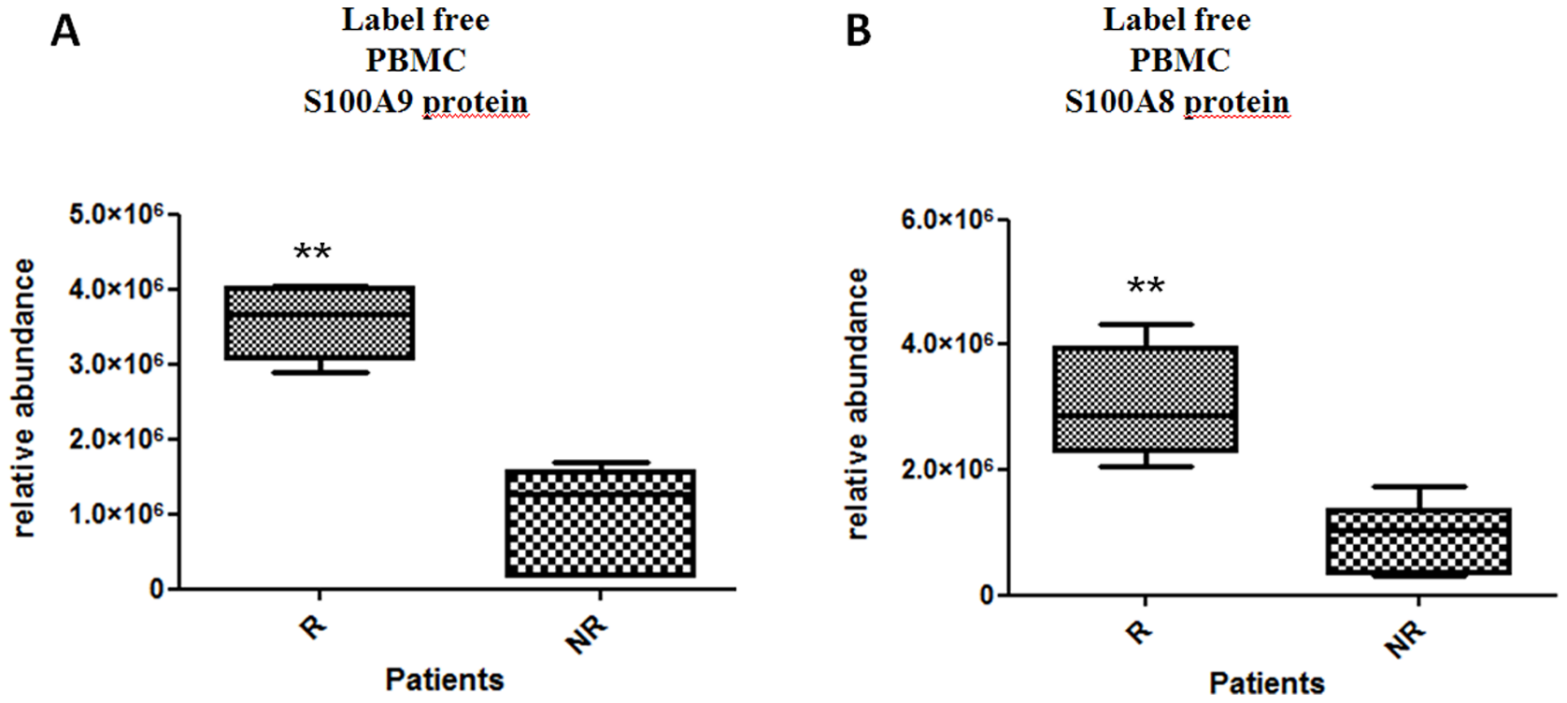
Baseline expression of S100A8 and S100A9 in PBMCs from RA patients according to the response/non-response status to the ETA/MTX combination in the first population. Relative quantification by mass spectrometry of S100A9 protein (A) and S100A8 protein (B) at baseline in responders (n = 3) versus non-responders (n = 3) to MTX/etanercept (significant difference is noted by asterisk, p<0.05; Mann-Whitney non-parametric test). Both S100A9 and S100A8 proteins accumulated in R patient (p-value  = 0.0022). The upper and lower bounds of each box indicate the 25^th^ and 75^th^ percentile respectively and heavy lines within the box represent the median. Whiskers are drawn to the min and max values.

From mass spectrometry intensities measurements, the S100A9 protein was expressed with a relative abundance of (3.56±0.20).10^6^ in R samples and (1.01±0.28).10^6^ in NR samples. The S100A8 protein was expressed with a relative abundance of (3.05±0.36).10^6^ in R samples and (9.51±2.25).10^5^ in NR samples. Interestingly, both S100A9 and S100A8 proteins, respectively quantified with 4 and 5 peptides, display a significant over-expression (p-value  = 0.0022) in R patients compared with NR (Fig. 2). For these two proteins, data revealed an abundance threshold able to separate the two populations under study. However, at this step, with a small number of patients, we do not claim that these proteins represent biomarkers, but rather they proposed an interesting increased abundance level in PBMCs isolated from R patients.

### Only S100A9 is over-expressed in the serum of responders

A second population of 22 RA patients was recruited to study the serum expression of S100A8 and S100A9 proteins, prior to MTX/ETA treatment (Population 2). Demographic, clinical and biological characteristics of the population are given in Table 1. According to the EULAR criteria, 12 patients were classified as R and 10 patients as NR after six months of ETA/MTX treatment. Indeed, the DAS28 score significantly decreased in R patients with a delta DAS28 of −2.57±0.18, whereas it remained unmodified in NR patients with a delta DAS28 of 0.17±0.17. To ascertain that those groups were unbiased, a statistical study was performed. Before treatment, four parameters (CRP, DAS28, ESR and HAQ) were slightly higher in R compared to NR but the differences were not significant (with p-values >0.05). Only two parameters were higher in NR (morning stiffness and pain) and the difference between these populations was again not significant. Concerning the immunological status, 80%/50% of NR patients and 75%/83% of R patients were RF/anti-CCP2 positive. Finally, the sex ratio was different in this population but women were more represented in both R and NR groups.

**Table 1:** Demographic, clinical and biological data of RA patients at baseline.

	Population 2 (serum)						
	Responders (n = 12)			Non responders (n = 10)			p-value
	Mean±SEM	median	min_max	mean±SEM	median	min_max	
**Age (years)**	51.1±3.8	50.89	22.5_69.5	58.7±4.4	63.81	26.69_74.85	0.12
**Sex (f/m)**	04/08	_		01/09	_		_
**Methotrexate (mg/week)**	15.0± 1.4	15.00	10.0_20.0	13.3±1.9	15.00	7.50_15.0	0.51
**Corticoids (mg/day)**	5.6±1.7	5.00	0_15	3.6±1.4	2.50	0.0_10.0	0.46
**Morning stiffness (min)**	47±16	30.00	0_180	62±27	30.00	10.0_240	0.62
**Pain (0–100 mm VAS)**	61.8±4.8	65.00	40.0_90.0	63.7±6.0	67.50	40.0_80.0	0.77
**ESR (mm/hour)**	27.3±6.2	22.00	3.0_76.0	22.0±5.2	18.00	11.0_56.0	0.51
**CRP (mg/l)**	20.6±7.9	11.00	2.0_71.2	9.5±5.8	5.50	1.0_44.0	0.072
**FR (UI/ml)**	137±52	51.00	0_621	370±250	81.50	0_2520	0.92
**Anti_CCP2 (UA/ml)**	84±32	45.00	0_400	89±48	7.50	0_400	0.31
**HAQ score (0_3)**	1.4±0.3	1.13	1.0_2.0	1.09±0.16	1.13	0.75_1.38	0.60
**DAS28**	4.17± 0.26	4.23	2.55_5.69	3.41±0.34	3.58	1.45_4.72	0.12
**DAS28 6 month**	1.61±0.16	1.58	0.69_2.48	3.59±0.32	3.90	1.98_4.64	0.0004
**ΔDAS28**	−2.57±0.18	−2.65	−3.27_−1.27	0.17±0.17	−0.052	−0.33_1.6	<0.0001

Values are expressed as mean ± SEM, and median with min and max values. This population included patients whose sera were collected. All differences between responders versus non-responders at baseline were non-significant (p-values >0.05, Mann-Whitney non-parametric test). Only DAS28 at 6 month and Delta DAS28 showed a significant difference between R and NR patients. CRP. C-reactive protein; DAS28. disease activity score at initiation of treatment; Δ DAS28. Difference between 6 months and baseline; ESR. erythrocyte sedimentation rate; HAQ. Health Assessment Questionnaire; VAS. visual analogue scale (patient's assessment of pain); RF. rheumatoid factor; anti-CCP2. anti-cycliccitrullinated peptide antibodies.

**Figure 3:**
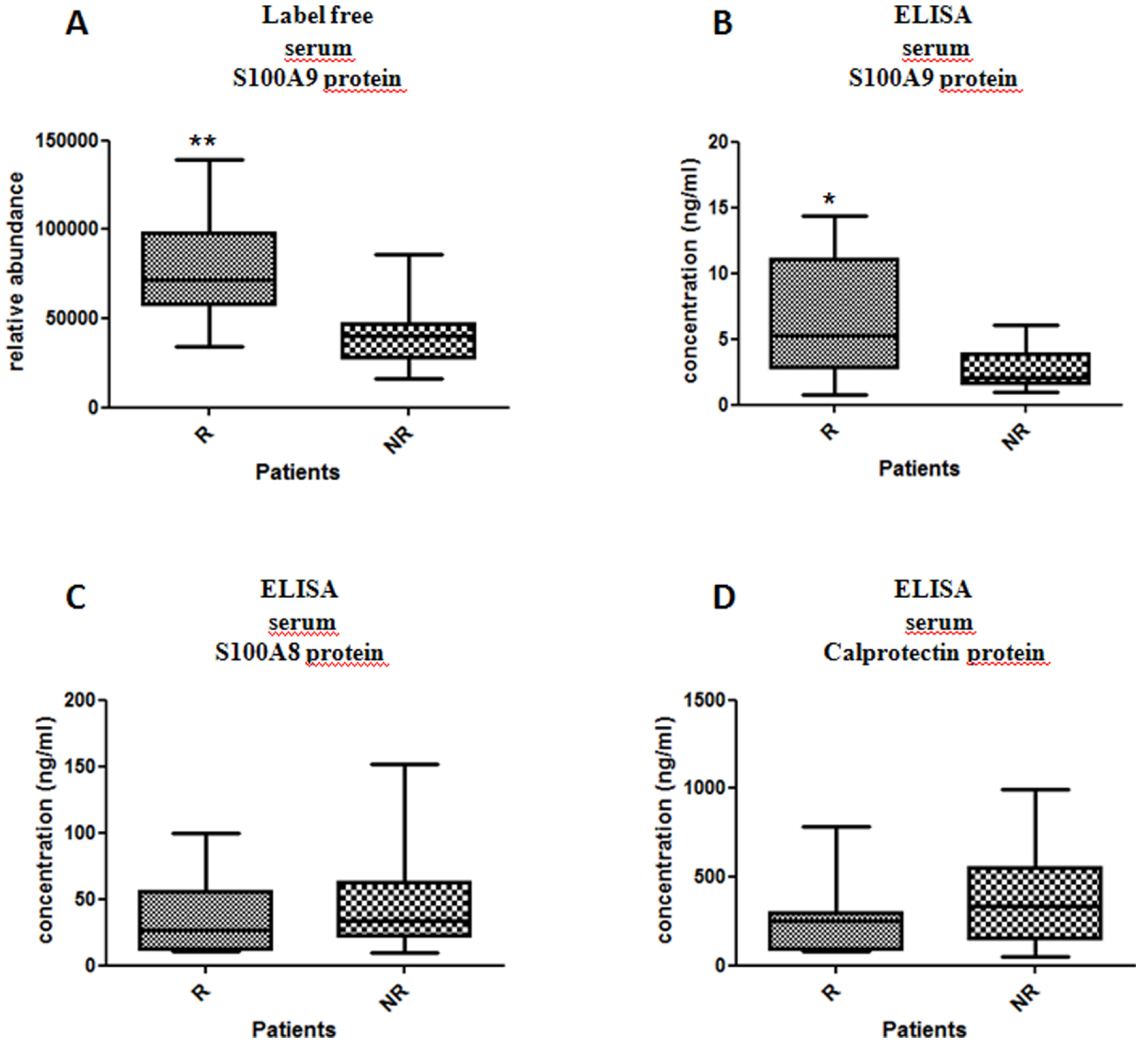
Over-expression of the S100A9 protein in baseline serum from responder patients to the MTX/ETA combination according to 2 different approaches in the second population. Relative quantification of serum S100A9 protein at baseline in R (n = 12) versus NR (n = 10) by mass spectrometry (A). This result showed an over-expression of S100A9 protein in R patient (p-value  = 0.0022). Serum ELISA absolute quantification of S100A9 (B), S100A8 (C) and calprotectin (D) at baseline in responders versus non-responders. No significant difference in the expression of S100A8 protein and calprotectin was observed between R and NR (p-value  = 0.75 and 0.32, respectively). Only S100A9 showed an over-expression in R patients (p-value  = 0.023).

From mass spectrometry investigations on sera protein extracts, we also measured the peptide relative abundances corresponding to proteins S100A8 and S100A9. For the S100A9 protein, 3 unique peptides were consistently and significantly over-expressed in R patients (data not shown). After normalization across all samples, the relative abundance of S100A9 at baseline was compared between R and NR patients using the unpaired T test (Fig. 3A). These results demonstrated a significant over-expression of S100A9 in the R group (p-value  = 0.0023). Concerning the S100A8 protein, 3 peptides allowed the protein identification. However, from statistical analysis, two of them were not differently expressed between R and NR groups. And with only one remaining peptide, our quality criteria were not fulfilled to assess a good evaluation of abundance.

### Confirmation of S100A9 over-expression using ELISA

To confirm independently these results, an ELISA approach was developed to achieve absolute quantification for S100A9 and S100A8 proteins but also for calprotectin from sera samples of patients belonging to the second population. Calprotectin was also tested because it corresponds to a heterodimeric complex of S100A8 and S100A9 suggested to be the biologically active form. These results demonstrate that the abundances of S100A8 and calprotectin were similar in R and NR patients (respectively p-value  = 0.751 and p-value  = 0.315). Using this orthogonal approach, S100A9 also displayed a significant over-expression in R patients (p-value  = 0.023; Fig. 3B and Table 2).

**Table 2:** Absolute and relative abundance of S100 proteins in serum samples.

	Population 2 (serum)		
	Responders (n = 12)	Non Responders (n = 10)	p-value
**S100A9 (Label free)**	(7.7±0.8).10^4^	(4.1±0.6).10^3^	0.0022**
**S100A9 (ELISA)**	6.5±1.4	2.7±0.5	0.029 *
**S100A8 (ELISA)**	37±9	47 ±13	0.75
**Calprotectin (ELISA)**	510±130	770±210	0.32

Relative quantification of serum S100A9 protein at baseline in R versus NR by mass spectrometry measurements and absolute quantification of serum S100 proteins at baseline in responders versus non-responders by ELISA (significant difference is indicated by an asterisk; p-value <0.05; Mann Whitney non-parametric test).

**Figure 4:**
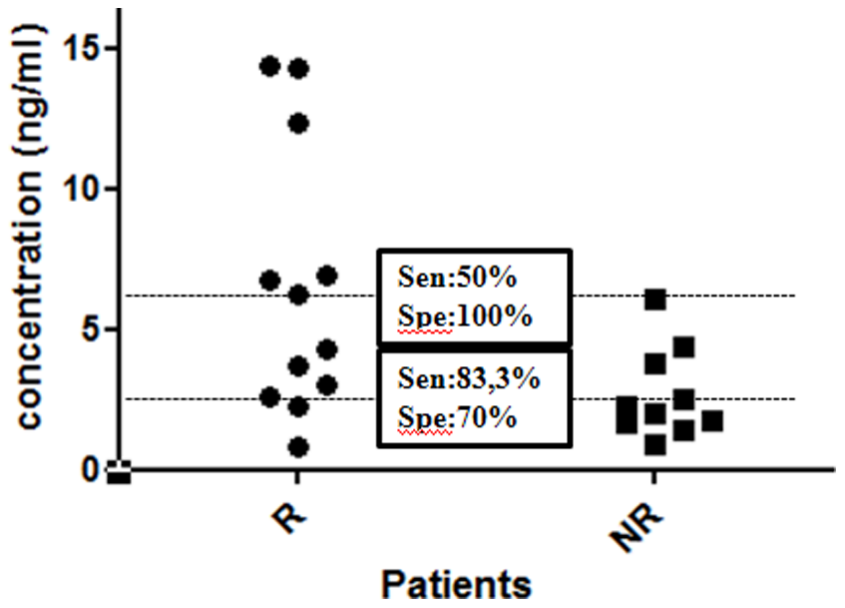
Thresholds of serum S100A9 concentration able to identify non-responders patients. Absolute quantification by ELISA of serum S100A9 protein in responders versus non-responders prior to MTX/ETA initiation. The calculated thresholds resulting from ROC analyses are also given with the corresponding sensitivities (Sen) and specificities (Spe). Circles and squares represent individual points for R (n = 12) and NR (n = 10) patients respectively.

This absolute quantification by ELISA for the S100A9 protein allowed us to determine a concentration threshold (2.59 ng/ml) associated to a right classification in the R and NR subsets, using ROC analysis. This threshold yielded discrimination between R from NR with a sensitivity of 83.3% and a specificity of 70% (Fig. 4). In addition, it was possible to identify all non-responders patients of this cohort using a threshold of 6.17 ng/ml.

### S100A9 is an independent predictor of MTX/ETA responsiveness

The relationship between the different parameters measured prior to ETA/MTX initiation and the response at 6 months was also evaluated (Table 3). The results show that baseline levels of CRP, ESR, RF and anti-CCP2 are not correlated to the response observed at 6 months. Only the relative abundance of S100A9 showed a significant correlation with delta DAS28. Moreover, the absolute quantification of S100A9 showed a correlation with delta DAS28, this link tending to be significant (p-value  = 0.056). Thus, the response of RA patients to MTX/ETA was not influenced by markers of inflammation or autoantibody status but only by the baseline expression of S100A9 in this second cohort.

**Table 3:** Relationship between baseline levels of several potential markers measured prior to MTX/ETA initiation and the degree of response over the 6 months follow-up in the second population.

	DAS28 6 month	p-value	Δ DAS28	p-value
**CRP**	−0.25	0.37	−0.25	0.37
**ESR**	0.25	0.30	−0.20	0.41
**FR**	0.16	0.53	0.16	0.54
**Anti-CCP**	0.03	0.91	0.16	0.59
**S100A9 Label free**	−0.51	0.016*	−0.56	0.006**
**S100A9 ELISA**	−0.41	0.057	−0.41	0.056

Correlations were analyzed by using Spearman's rank correlations. Linear regression analysis was performed between (i) CRP levels, ESR values, abundances of protein S100A9, RF titers, anti-CCP titers and (ii) DAS28 at 6 month or ΔDAS28 (significant difference is noted by an asterisk).

## Discussion

This study was conducted to identify potential serum protein biomarkers able to predict the response to the ETA/MTX combination before treatment initiation. In this respect, we have not studied the expression fluctuations of the different markers during the first weeks of treatment. Indeed, in clinical practice, the objective is to avoid initiation of an inappropriate treatment and to introduce the one likely to be the most effective among all biological agents available in first-line after non-biological DMARD failure. In the present study, the degree of response to the ETA/MTX combination in RA patients was shown to be correlated to both PBMCs and serum levels of S100A9 protein, measured prior to treatment initiation. Finally, S100A9 protein acts as an independent predictor when compared to standard biomarkers (CRP, auto-antibodies).

Previously restricted to the simple identification of protein sample, the recent progresses in mass spectrometry (including resolution and accuracy) allow the quantification of proteins within complex matrices. The so-called "label free" approach enables protein relative quantification in several samples based on the comparison of intensities of peptide ion currents observed during LC separations [16]. This challenging technology is accurate and robust enough to estimate protein ratios after adequate statistical processing. Although initiated to draw a global picture, this work was focused on two proteins of interest, namely S100A8 and S100A9 proteins. These two proteins are constitutively expressed in neutrophils and monocytes [19] but also in macrophages during acute or chronic inflammation. They are associated with various infectious or inflammatory diseases [20]–[24]. Recently, some studies have shown that biological therapies modulate the expression of S100 proteins. Indeed, the expression of several genes, including those coding for S100A12 and S100A8 proteins was lower in PBMCs after treatment with etanercept or adalimumab [25]. Besides, the level of soluble calprotectin was shown to decrease and to be associated to ultrasonographic synovitis scores in RA patients treated by adalimumab [26]. Thus, calprotectin might be of additional value in the assessment of RA patients under biologic treatment. However, even though levels of S100 proteins appear to be influenced by TBAs, their theranostic value has never been demonstrated until now. So far, these proteins have only been identified as diagnostic [9], [10] and prognostic [13] markers of RA. Furthermore, these proteins are found at high concentrations in the serum of RA patients. A high correlation is observed between flares and elevated concentration of these proteins, making the latter interesting inflammatory markers, reflecting the disease activity [27].

Here, proteomic investigations were firstly performed with PBMCs because these cells and several cytokines produced by them have a pivotal role in RA pathogenesis and are targeted by ETA. Specifically, in the context of RA, PBMCs constitute an advantageous surrogate tissue as they allow for screening in any subject, whereas synovium is only accessible through an invasive procedure. Additionally, proteomic investigations on PBMCs benefit from a reduced dynamic range of protein concentration compared to serum. The results of this first part of the study demonstrated a similar over-expression of S100A8 and S100A9 proteins in the cellular PBMC proteome, where they might appear as a heterodimeric complex. Thereafter, we investigated the "theranostic" potential of S100 proteins in sera from R and NR patients to ETA/MTX combination. In contrast to PBMC investigations, this study only revealed a significant over-expression of the S100A9 protein in R patients, confirmed by ELISA absolute quantification. Conversely, ELISA revealed a similar expression of S100A8 and calprotectin in both R and NR groups.

Thus, in the present study, PBMC S100A8 over-expression associated to MTX/ETA response was not observed when the protein concentration was measured from the serum samples. These data suggest that S100A8 may not be as strong biomarker as S100A9. Furthermore, it should be reminded that the S100 proteins do not possess any signal peptide that allows them to be secreted by the classical pathway of the endoplasmic reticulum and the golgi apparatus. In relation to our observations, different stimuli may promote a significant release of S100A8/A9 as lipopolysaccharide, granulocyte-macrophage colony-stimulating factor, interleukin-1 beta, whereas other stimuli such as pokeweed mitogen do not induce release of S100A8[28]. This discordant result between PBMC and sera can be also alternatively attributed to the small sampling for PBMC analysis.

Even though S100A9 protein has a less significant over-expression in responder sera than in PBMC, it was possible to determine a concentration threshold able to identify non-responders with appropriate sensitivity and specificity. Interestingly, a threshold related to a systematic non-response to the MTX/ETA combination was highlighted in the cohort. This information is of particular interest for clinicians since this combination will not be prescribed to patients exhibiting serum S100A9 concentrations above this cut-off. However, using this threshold, the sensitivity is reduced to 50%.

In the literature, two reports attempted to identify predictive biomarkers of response to ETA. However, none of them highlighted a possible interest for S100 proteins [29], [30]. Noteworthy, the work of Fabre *et al*. showed that elevated serum level of monocyte chemo-attractant protein-1 (MCP-1) predicts a good response to ETA treatment. MCP-1 regulates the migration and infiltration of monocytes, CD4 memory T lymphocytes and NK cells. Interestingly, S100 proteins were also related to cell migration via the remodelling of the cytoskeleton [31]. Thus, these two proteins, namely S100A9 and MCP-1, involved in monocyte migration may be key actors of the response to the soluble form of the TNF-alpha receptor.

## Conclusion

Herein, the potential theranostic value of S100A9 protein was demonstrated both in PBMCs and serum collected prior to MTX/ETA initiation. This potential biomarker was identified using an innovative proteomic methodology and was confirmed using a more traditional but equally robust ELISA approach. Due to the limited number of patients, this study constitutes a first piece of evidence requiring to be confirmed with a larger cohort. In this study, S100A9 was the only marker correlated with the response to treatment since both parameters of inflammation and autoantibodies did not yield to such a relationship. This protein might be used to guide physicians in the prescription of first line biotherapy after MTX failure. Nevertheless, monitoring of a single biomarker is often not enough robust to predict the response to treatment and it is of major importance to have access to a combination of biomarkers, such as S100A9 associated with known markers (e.g. MCP-1) or with protein markers to be discovered by further investigations.

## References

[1] Brennan FM, Maini RN, Feldmann M (1998) Role of pro-inflammatory cytokines in rheumatoid arthritis. Springer Semin Immunopathol 20:133–147. 9836373 10.1007/BF00832003

[2] Singh JA, Christensen R, Wells GA, Suarez-Almazor ME, Buchbinder R, et al (2009) A network meta-analysis of randomized controlled trials of biologics for rheumatoid arthritis: a Cochrane overview. CMAJ 181:787–796. 19884297 10.1503/cmaj.091391 PMC2780484

[3] Bathon JM, Martin RW, Fleischmann RM, Tesser JR, Schiff MH, et al (2000) A comparison of etanercept and methotrexate in patients with early rheumatoid arthritis. N Engl J Med 343:1586–1593. 11096165 10.1056/NEJM200011303432201

[4] Keystone EC, Kavanaugh AF, Sharp JT, Tannenbaum H, Hua Y, et al (2004) Radiographic, clinical, and functional outcomes of treatment with adalimumab (a human anti-tumor necrosis factor monoclonal antibody) in patients with active rheumatoid arthritis receiving concomitant methotrexate therapy: a randomized, placebo-controlled, 52-week trial. Arthritis Rheum 50:1400–1411. 15146409 10.1002/art.20217

[5] Keystone EC, Schiff MH, Kremer JM, Kafka S, Lovy M, et al (2004) Once-weekly administration of 50 mg etanercept in patients with active rheumatoid arthritis: results of a multicenter, randomized, double-blind, placebo-controlled trial. Arthritis Rheum 50:353–363. 14872476 10.1002/art.20019

[6] Lequerre T, Jouen F, Brazier M, Clayssens S, Klemmer N, et al (2007) Autoantibodies, metalloproteinases and bone markers in rheumatoid arthritis patients are unable to predict their responses to infliximab. Rheumatology (Oxford) 46:446–453. 16899502 10.1093/rheumatology/kel262

[7] Sinz A, Bantscheff M, Mikkat S, Ringel B, Drynda S, et al (2002) Mass spectrometric proteome analyses of synovial fluids and plasmas from patients suffering from rheumatoid arthritis and comparison to reactive arthritis or osteoarthritis. Electrophoresis 23:3445–3456. 12373775 10.1002/1522-2683(200210)23:19<3445::AID-ELPS3445>3.0.CO;2-J

[8] Uchida T, Fukawa A, Uchida M, Fujita K, Saito K (2002) Application of a novel protein biochip technology for detection and identification of rheumatoid arthritis biomarkers in synovial fluid. J Proteome Res 1:495–499. 12645617 10.1021/pr025531w

[9] de Seny D, Fillet M, Meuwis MA, Geurts P, Lutteri L, et al (2005) Discovery of new rheumatoid arthritis biomarkers using the surface-enhanced laser desorption/ionization time-of-flight mass spectrometry ProteinChip approach. Arthritis Rheum 52:3801–3812. 16320331 10.1002/art.21607

[10] Baillet A, Trocme C, Berthier S, Arlotto M, Grange L, et al (2010) Synovial fluid proteomic fingerprint: S100A8, S100A9 and S100A12 proteins discriminate rheumatoid arthritis from other inflammatory joint diseases. Rheumatology (Oxford) 49:671–682. 20100792 10.1093/rheumatology/kep452

[11] Drynda S, Ringel B, Kekow M, Kuhne C, Drynda A, et al (2004) Proteome analysis reveals disease-associated marker proteins to differentiate RA patients from other inflammatory joint diseases with the potential to monitor anti-TNFalpha therapy. Pathol Res Pract 200:165–171. 15237925 10.1016/j.prp.2004.02.011

[12] Hammer HB, Odegard S, Fagerhol MK, Landewe R, van der Heijde D, et al (2007) Calprotectin (a major leucocyte protein) is strongly and independently correlated with joint inflammation and damage in rheumatoid arthritis. Ann Rheum Dis 66:1093–1097. 17234650 10.1136/ard.2006.064741 PMC1954700

[13] Liao H, Wu J, Kuhn E, Chin W, Chang B, et al (2004) Use of mass spectrometry to identify protein biomarkers of disease severity in the synovial fluid and serum of patients with rheumatoid arthritis. Arthritis Rheum 50:3792–3803. 15593230 10.1002/art.20720

[14] Andres Cerezo L, Mann H, Pecha O, Plestilova L, Pavelka K, et al (2011) Decreases in serum levels of S100A8/9 (calprotectin) correlate with improvements in total swollen joint count in patients with recent-onset rheumatoid arthritis. Arthritis Res Ther 13:R122. 21791097 10.1186/ar3426 PMC3239361

[15] Lequerre T, Gauthier-Jauneau AC, Bansard C, Derambure C, Hiron M, et al (2006) Gene profiling in white blood cells predicts infliximab responsiveness in rheumatoid arthritis. Arthritis Res Ther 8:R105. 16817978 10.1186/ar1990 PMC1779405

[16] Old WM, Meyer-Arendt K, Aveline-Wolf L, Pierce KG, Mendoza A, et al (2005) Comparison of label-free methods for quantifying human proteins by shotgun proteomics. Mol Cell Proteomics 4:1487–1502. 15979981 10.1074/mcp.M500084-MCP200

[17] Arnett FC, Edworthy SM, Bloch DA, McShane DJ, Fries JF, et al (1988) The American Rheumatism Association 1987 revised criteria for the classification of rheumatoid arthritis. Arthritis Rheum 31:315–324. 3358796 10.1002/art.1780310302

[18] van Gestel AM, Prevoo ML, van 't Hof MA, van Rijswijk MH, van de Putte LB, et al (1996) Development and validation of the European League Against Rheumatism response criteria for rheumatoid arthritis. Comparison with the preliminary American College of Rheumatology and the World Health Organization/International League Against Rheumatism Criteria. Arthritis Rheum 39:34–40. 8546736 10.1002/art.1780390105

[19] Hessian PA, Edgeworth J, Hogg N (1993) MRP-8 and MRP-14, two abundant Ca(2+)-binding proteins of neutrophils and monocytes. J Leukoc Biol 53:197–204. 8445331

[20] Barthe C, Figarella C, Carrere J, Guy-Crotte O (1991) Identification of 'cystic fibrosis protein' as a complex of two calcium-binding proteins present in human cells of myeloid origin. Biochim Biophys Acta 1096:175–177. 2001432 10.1016/0925-4439(91)90057-g

[21] Benoit S, Toksoy A, Ahlmann M, Schmidt M, Sunderkotter C, et al (2006) Elevated serum levels of calcium-binding S100 proteins A8 and A9 reflect disease activity and abnormal differentiation of keratinocytes in psoriasis. Br J Dermatol 155:62–66. 16792753 10.1111/j.1365-2133.2006.07198.x

[22] Summerton CB, Longlands MG, Wiener K, Shreeve DR (2002) Faecal calprotectin: a marker of inflammation throughout the intestinal tract. Eur J Gastroenterol Hepatol 14:841–845. 12172403 10.1097/00042737-200208000-00005

[23] Haga HJ, Brun JG, Berntzen HB, Cervera R, Khamashta M, et al (1993) Calprotectin in patients with systemic lupus erythematosus: relation to clinical and laboratory parameters of disease activity. Lupus 2:47–50. 8485559 10.1177/096120339300200108

[24] Sweet SP, Denbury AN, Challacombe SJ (2001) Salivary calprotectin levels are raised in patients with oral candidiasis or Sjogren's syndrome but decreased by HIV infection. Oral Microbiol Immunol 16:119–123. 11240866 10.1034/j.1399-302x.2001.016002119.x

[25] Meugnier E, Coury F, Tebib J, Ferraro-Peyret C, Rome S, et al (2011) Gene expression profiling in peripheral blood cells of patients with rheumatoid arthritis in response to anti-TNF-alpha treatments. Physiol Genomics 43:365–371. 21266503 10.1152/physiolgenomics.00127.2010

[26] Hammer HB, Fagerhol MK, Wien TN, Kvien TK (2011) The soluble biomarker calprotectin (an S100 protein) is associated to ultrasonographic synovitis scores and is sensitive to change in patients with rheumatoid arthritis treated with adalimumab. Arthritis Res Ther 13:R178. 22029973 10.1186/ar3503 PMC3308113

[27] Foell D, Frosch M, Sorg C, Roth J (2004) Phagocyte-specific calcium-binding S100 proteins as clinical laboratory markers of inflammation. Clin Chim Acta 344:37–51. 15149869 10.1016/j.cccn.2004.02.023

[28] Lugering N, Kucharzik T, Lugering A, Winde G, Sorg C, et al (1997) Importance of combined treatment with IL-10 and IL-4, but not IL-13, for inhibition of monocyte release of the Ca(2+)-binding protein MRP8/14. Immunology 91:130–134. 9203976 10.1046/j.1365-2567.1997.00221.x PMC1364045

[29] Fabre S, Dupuy AM, Dossat N, Guisset C, Cohen JD, et al (2008) Protein biochip array technology for cytokine profiling predicts etanercept responsiveness in rheumatoid arthritis. Clin Exp Immunol 153:188–195. 18549443 10.1111/j.1365-2249.2008.03691.x PMC2492901

[30] Hueber W, Tomooka BH, Batliwalla F, Li W, Monach PA, et al (2009) Blood autoantibody and cytokine profiles predict response to anti-tumor necrosis factor therapy in rheumatoid arthritis. Arthritis Res Ther 11:R76. 19460157 10.1186/ar2706 PMC2714123

[31] Vogl T, Ludwig S, Goebeler M, Strey A, Thorey IS, et al (2004) MRP8 and MRP14 control microtubule reorganization during transendothelial migration of phagocytes. Blood 104:4260–4268. 15331440 10.1182/blood-2004-02-0446

